# Antitumor activity of gamma-irradiated *Rosa canina L.* against lung carcinoma in rat model: a proposed mechanism

**DOI:** 10.1186/s12906-025-04813-1

**Published:** 2025-02-28

**Authors:** Omayma A. R. Abo-Zaid, Fatma S. M. Moawed, Eman S. Eldin, Mostafa A. Farrag, Esraa S. A. Ahmed

**Affiliations:** 1 Biochemistry and Molecular Biology Department, Faculty of Vet. Med, Benha University, Benha, Egypt; 2 Health Radiation Research, National Center for Radiation Research and Technology, Egyptian Atomic Energy Authority, Cairo, Egypt; 3 Radiation Biology , National Center for Radiation Research and Technology, Egyptian Atomic Energy Authority, Nasr City, Cairo 11787 Egypt

**Keywords:** Lung cancer, *Rosa canina*, *Cisplatin*, PARP-1, TLR2/MyD88/ TRAF6/NF-κB, PI3K/AKT/mTOR, Autophagy, Apoptosis

## Abstract

**Background:**

Lung cancer is one of the most prevalent malignancies globally and is the leading cause of cancer-related mortality. Although cisplatin is a widely utilized chemotherapeutic agent, its clinical efficacy is often hampered by significant toxicity and undesirable side effects. Rosa canina, a medicinal plant, has demonstrated a range of beneficial biological activities, including anti-inflammatory, anticancer, immunomodulatory, antioxidant, and genoprotective effects.

**Methods:**

This study aimed to investigate the potential of Rosa canina to enhance the anticancer efficacy of cisplatin in a dimethyl benz(a)anthracene-induced lung cancer model using female rats. The animals were administered Rosa canina, cisplatin, or a combination of both treatments. The expression levels of critical signaling molecules were evaluated, including phosphoinositide-3-kinase (PI3K), Akt, mammalian target of rapamycin (mTOR), cleaved poly (ADP-ribose) polymerase (PARP-1), myeloid differentiation factor 88 (MyD88), and tumor necrosis factor receptor-associated factor (TRAF), in addition to various autophagic markers. Furthermore, we assessed the levels of toll-like receptor 2 (TLR2), nuclear factor kappa B (NF-κB), and apoptotic markers in lung tissue, complemented by histopathological examinations.

**Results:**

The combined treatment of Rosa canina extract and cisplatin significantly inhibited lung cancer cell proliferation by downregulating PARP-1 and the TLR2/MyD88/TRAF6/NF-κB signaling pathway, as well as the PI3K/Akt/mTOR pathway. Moreover, this combination therapy promoted autophagy and apoptosis, evidenced by elevated levels of autophagic and apoptotic markers.

**Conclusion:**

Overall, the findings of this study suggest that Rosa canina enhances the anticancer effects of cisplatin by inhibiting cancer cell proliferation while simultaneously inducing autophagy and apoptosis. Thus, Rosa can be used as adjuvant to cisplatin chemotherapy to overcome its limitations which may be considered a new approach during lung cancer treatment strategy.

**Graphical Abstract:**

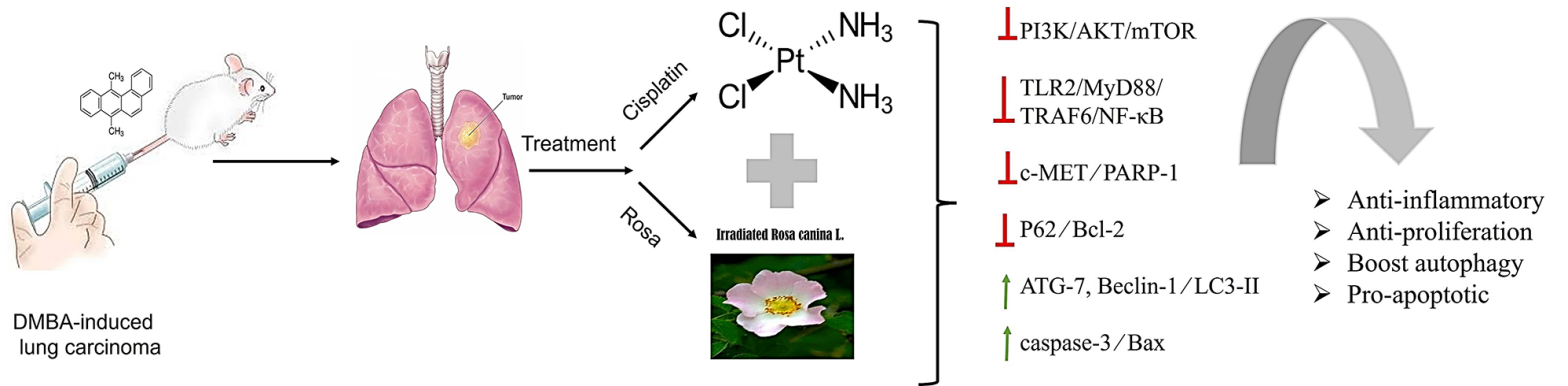

## Background

Lung cancer is one of the most common malignancies worldwide with the highest mortality rates accounting for 25% of all cancer deaths globally based on the latest data released by the IARC of the WHO [1, 2]. It is reported as the fifth most common cancer in Egypt with an estimated incidence of 5.1% and 5-year prevalence of 2.9 and the fourth with a high rate of mortality of 7.1% [3]. Many risk factors contribute to lung carcinogenesis encompassing environmental and air pollution, smoking, radiation, chronic lung diseases, family history and genetic mutations. Smokers are four to ten times more likely to develop lung cancer than non-smokers [4, 5]. Environmental and air pollution related to modernization, industrial activities and traffic deliver a heterogeneous mixture of chemical toxicants and carcinogens such as polycyclic aromatic hydrocarbons (PAHs) [6].

One of the commonly known PAHs is 7,12-dimethylbenz (a) anthracene (DMBA), which is found in high concentrations in car exhaust, cigarette smoke, medicines, dyes, plastics, pesticides, aerosols and others. Widely, it was reported that DMBA has an adverse mechanism for health hazards during its metabolism into different reactive metabolic intermediates such as 3,4-diol-1,2-epoxide which covalently binds to purine bases leading to DMBA-DNA adduct coupled with the generation of free radical and reactive oxygen species (ROS) leading to oxidative stress and inflammation [7, 8]. Moreover, under a defective and dysfunctional DNA repair system these DNA adducts potentiate mutations and genetic instability with subsequent promotion of carcinogenesis [9]. Collectively, DMBA was used as a broad carcinogen in experimental animals that induce various cancer models including skin, mammary, oral, lymphoid lung, ovarian cancer, leukemia and other neoplasms [10, 11].

Chemotherapy is a common strategy for the treatment of various types of lung cancer and cisplatin is the most predominantly used chemotherapeutic agent [12]. It can interact with DNA causing DNA damage and activating apoptotic signaling pathways. However, despite its widespread application, its clinical efficacy is diminished due to the drug resistance subsequent to increasing doses coupled with toxicity and side effects [13].

Phytochemical compounds found in many medicinal plants are natural, and abundant, with lower toxicity, high efficiency, and the ability to target multi-pathways [14]. It was reported that natural products have anticancer potential through modulation of many dysregulated pathways involved in cancer initiation, development and progression which eventually hindered cell proliferation, angiogenesis, and metastasis and promoted apoptosis [15, 16]. Interestingly, about 47% of antitumor drugs are produced from natural compounds. Additionally, they can act as chemosensitizers [17], improving the efficiency of many chemotherapeutic agents with a potential propensity to minimize dosage frequency, side effects, toxicity and resistance of the chemotherapeutic agents [18, 19]. Furthermore, Yuwen et al. [20] exhibited that using natural products and phytochemicals in synergy with cisplatin against lung cancer augmented its potency via enhancing its antineoplastic activity.

*Rosa canina (R. canina)* or Rosehip (RH) is a medicinal plant that belongs to the Rosacea family. Regarding the safety profile (absence of toxicity and side effects) it has been effectively used in complementary medicine owing to its biological potencies [21]. These obvious beneficial health potentials are attributed to the abundance of various bioactive compounds vitamin C (ascorbic acid), other (B, K), phospholipids, polyphenolic molecules, macro- and micro-elements and minerals, polysaccharides, flavonoids, carotenoids and polyunsaturated fatty acids [22]. Furthermore, it has been demonstrated that besides its immunomodulatory, pain modulation, anti-diabetic, anti-hyperlipidemic, neuroprotective, genoprotective and antioxidant properties, *R. canina* displayed a beneficial anti-inflammatory effect by inhibiting NF-κB activation [23, 24] in addition to its anti-cancer and chemotherapeutic effect against different cancer cell lines including colon, lung and prostate [21, 25].

Despite the absence of an oncological clinical trial that uses *R canina* extract in humans, its potential antitumor properties have also been tested in various experimental studies [26]. Fujii et al. [27] showed that rose hips inhibited melanogenesis in mouse melanoma cells and guinea pig skin by reducing the intracellular tyrosinase activity owing to the presence of proanthocyanidins. Both Jimenez et al. [28] and Turan et al. [29] confirmed the antiproliferative effect of *R. canina* extracts on colon cancer (Caco-2) and (WiDr) cell lines respectively which was attributed to triggering apoptosis and arresting the cell cycle at the S phase. Moreover, Kilinc et al. [30] reported that *R. canina* extract has an antiproliferative and apoptotic effect on human lung (A549) and prostate (PC-3) cancer cells by inducing cell cycle arrest at the G_1_ phase, reducing mitochondrial membrane potential (MMP) and increasing caspase activity in these cells. Another study demonstrated the potential anti-breast cancer effect of *R canina* ethanolic extract against MCF-7 and MDA-MB-468 cancer cell lines without affecting the normal cells *via* promoting cell lysis by the apoptosis pathway [31].

Interestingly, it was illustrated that the gamma irradiation of dried RH at doses of 3–10 kGy does not significantly change most of its bioactive compounds (sugars, organic acids, fatty acids, *β*-carotene, and total polyphenols) ensuring the safety of gamma irradiation technology on sterilization and decontamination of foodstuffs [32]. Collectively, this study aims to evaluate the potential antitumor efficacy of *Rosa canina* against DMBA-induced lung cancer model in female rats and whether it can enhance the chemotherapeutic activity of cisplatin.

## Material and method

### Materials

7, 12–dimethylbenz (a) anthracene (DMBA), cisplatin and all reagents used in this study were purchased from Sigma-Aldrich (St. Louis, Missouri, USA). *Rosa canina L*. fruits were purchased from a local market store.

### Preparation of irradiated *R. canina* extract

Briefly, according to the method of Moustafa et al. [33] 500 g from clean dried and ground rosa fruits packaged in polyethylene pouches were exposed to 1 k Gy γ- radiation by ^60^Co from the Gamma Chamber 4000 unit at the National Center for Radiation Research and Technology (NCRRT, Atomic Energy Authority, Egypt). The dose rate at the time of the experimentation was 2.3 kGy/h. after that, in a Soxhlet device, the irradiated rosa was soaked in aqueous ethanol (70%) for 48 h at room temperature. The ethanolic extract was filtered and the solvent was evaporated from the filtrate under reduced pressure in a vacuum rotary evaporator (model RE52A, China) to obtain a concentrated brown residue. This residue was not completely dried but became concentrated. Herein, a cyclomixer was used to disperse the obtained ethanolic extract residue in double-distilled water to create a more uniform solution for bioactivity testing.

### Animals

Thirty female Swiss albino rats (100–120 g) at the age of 6–8 weeks were obtained from (the Nile Company for Pharmaceuticals & Chemical Industries S.A.E. (Egypt)) Institutional animal house in standard laboratory conditions and placed in clean plastic cages at 25 ± 2 °C, and a constant 12 h light/ dark cycle with free access to a pellet diet and water ad libitum during the study.

### Ethical approval

The handling of the experimental animals involved in this study was approved by the Use Committee Research Ethic Board of Benha University, Faculty of Veterinary Medicine (BUFVTM01-03-24).

### Experimental animal model

To examine the antitumor effect of cisplatin and/or *R. canina*, 7,12-dimethyl benz [a] anthracene (DMBA) was used to conduct a lung carcinoma model in rats. The female rats were injected intravenously with three doses of DMBA (35 mg/kg body weight) dissolved in dimethyl sulfoxide at biweekly intervals [34].

### Experimental groups

After a week of accommodation, the rats were randomly categorized into five equal groups (6 each) as follows.

1. Control Group: Normal rats served as control.
2. DMBA Group: rats were injected with DMBA to induce lung carcinoma as mentioned above in the experimental animal model and left for one month before any treatment.
3. DMBA + Rosa Group: rats were injected with DMBA to induce lung carcinoma and left for one month before any treatment then they were gavaged with ethanolic extract of irradiated Rosa at a dose of 500 mg/kg body weight [33] daily for four weeks.
4. DMBA + CIS: rats were injected with DMBA to induce lung carcinoma and left for one month before any treatment then they were treated with cisplatin (CIS) at a dose of 2.5 mg/kg [35] intraperitoneally once/week for four weeks.
5. DMBA + CIS + Rosa Group: rats were injected with DMBA to induce lung carcinoma and left for one month before any treatment then they were treated with cisplatin (CIS) and Rosa as mentioned above.

At the end of the experimental period, the animals were anesthetized with the recommended anesthetic dose of urethane (1.0–1.2 g/kg) intraperitoneally [36]. The rats were humanly euthanized by the removal and excision of the lung tissues immediately. Lung tissues were washed with ice-cold saline and divided into two parts. The first part was preserved in a 10% buffered formalin-saline solution for the histopathological examinations, while the other part was stored at − 80 ^◦^C for further biochemical analysis.

### Histopathological examinations

The lung tissues were fixed in a 10% buffered formalin-saline solution, processed, embedded in paraffin, sectioned at 4–6 μm thickness and stained with hematoxylin and eosin (H&E) according to the Bancroft et al. method [37]. The response of tumor mass to treatment was divided into the following: Grade I a, marginal or no regression; Grade I b, morphologic evidence of therapy-induced changes but > 10% residual tumor; Grade II a, extensive response but with residual tumor *≤* 10%; and Grade II b, pathologic complete response [38].

### Quantitative real-time polymerase chain reaction (RT-PCR) analysis

To determine the mRNA expression of phosphatidylinositol 3 kinase (Pi3k), Protein kinase B (Akt), mammalian target of rapamycin (Mtor), autophagy-related genes (Atg), Beclin-1, Microtubule-associated protein 1 A/1B-light chain 3 (Lc3ii), P62, cleaved poly ADP-ribose polymerase (Parp-1), myeloid differentiation factor 88 (Myd88) and tumor necrosis factor receptor-associated factor (Traf), RNA was extracted from 30 mg lung tissues using a total RNA Purification Kit following the manufacturer protocol (Thermo Scientific, Fermentas, #K0731). Complementary DNA (cDNA) synthesis was performed using Reverse Transcription Kits (Thermo Scientific, Fermentas, #EP0451). The isolated cDNA was amplified using SYBR Green qPCR Master Mix according to the manufacturer protocol (Thermo Scientific, USA, # K0221) and gene-specific primers with the sequences listed in Table 1. Using Sequence Detection Software (PE Biosystems, Massachusetts, USA), RT-PCRs were performed in a thermal cycler step one plus (Thermo Scientific, USA, # K0221). Relative expression of Pi3k, Akt, Mtor, Atg-7, Beclin-1, Lc3ii, P62, Parp, Myd88 and Traf-6 mRNA were calculated using the comparative Ct method according to Pfaffl method [39]. Calculations were performed by calculating the values of the D cycle threshold (DCt) by normalizing the average Ct value of each treatment compared to the endogenous control β-actin.

**Table 1:** Primer’s sequence used in qPCR

Gene	Forward primer (^/^5 ------ ^/^3)	Reverse primer (^/^5 ------ ^/^3)
*Pi3k*	AACACAGAAGACCAATACTC	TTCGCCATCTACCACTAC
*Akt*	GTGGCAAGATGTGTATGAG	CTGGCTGAGTAGGAGAAC
*Mtor*	GGTGGACGAGCTCTTTGTCA	AGGAGCCCTAACACTCGGAT
*Beclin-1*	CGGAATTCTATGGAAGGGTCTAAGACGTCC	CGGGATCCTCATTTGTTATAAAATTGTGAGGACA
*Atg7*	GCTGGTCTCCTTGCTCAAAC	CAGGGTGCTGGGTTAGGTTA
*P62*	TCCTGCAGACCAAGAACTATGACATCG	TCTACGCAAGCTTAACACAACTATGAGACA
*Lc3II*	CAGGATCCATGCCG TCC CAG AAG ACC	GTC CCT TTT TGC CTT GGT AG
*Parp-1*	CGGCACGAGAGGGAGGATGG	TGTCAGGCTGCCGGATGGAGT
*Myd88*	GAGATCCGCGAGTTTGAGAC	TTGTCTGTGGGACACTGCTC
*Traf6*	CAG TCC CCT GCA CATT	GAG GAG GCA TCG CAT
*β-actin*	AAGTCCCTCACCCTCCCAAAAG	AAGCAATGCTGTCACCTTCCC

### Enzyme-linked immunosorbent assay (ELISA) measurements

The levels of Toll-Like Receptor 2 (TLR2), Nuclear Factor Kappa B (NFkB), Bcl-2 Associated X Protein (BAX), B-Cell Leukemia/Lymphoma 2 (BCL-2), Caspase-3, mesenchymal-epithelial transition factor (c-Met) and Galectin-1 (Gal-1) were detected in lung tissue homogenates following the instructions of commercial ELISA kits from My BioSource Inc. (San Diego, California, USA).

### Statistical analysis

All data are expressed as the mean ± standard mean error (SEM). The Statistical analysis of the results was performed by one-way ANOVA and Bonferroni tests were used for the comparison between groups and the significance value was at *p* < 0.001. Statistical analysis was performed using the SPSS 20 software package (Analytical Software, USA) and all graphs were plotted by GraphPad Prism software version 8 (GraphPad Software, Inc., La Jolla, CA, USA).

## Results

### Histopathology

**Fig. 1:**
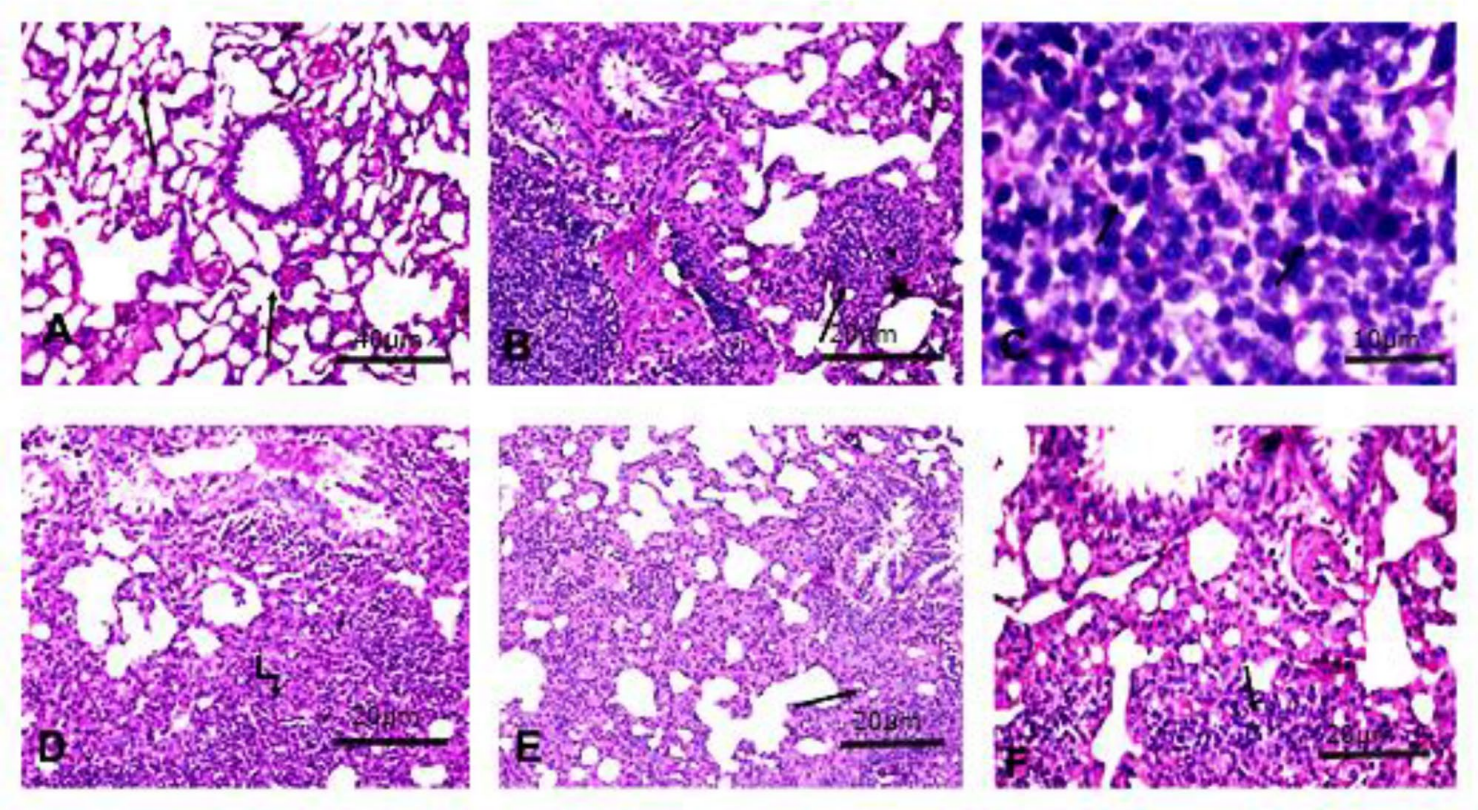
Photomicrograph of **Control** lung tissue (**A**): showing thin inter-alveolar septa **arrow** (x100), scale bar (40 μm). (**B**): **DMBA** lung section showing small cell carcinoma tumor mass and bronchi with fine stroma **arrow** (x200) scale bar (20 μm), (**C**) deeply basophilic nuclei and scanty cytoplasm **arrow** (x400) scale bar (10 μm). While (**D**): a photomicrograph of lung tissue of **DMBA + ROSA** group showing deeply basophilic neoplastic cells separated by delicate fibrous stroma **arrow** and apoptotic cells (x200) scale bar (20 μm). (**E**): represents lung tissue section of **DMBA + Cisplatin** group showing scattered neoplastic masses along lung lobules with thickening of alveolar septa **arrow** (x200) scale bar (20 μm). Furthermore, (**F**) represents lung section of **DMBA + ROSA + Cisplatin** group showing shrinkage of tumor mass **arrow** and apoptotic cells (x200) scale bar (20 μm)

The photomicrographs of the lung tissues section of control groups showed normal lung architecture with folded columnar epithelial cells of bronchiole, obvious alveolar sacs, normal pulmonary vessels and normal fibrous tissue distribution. The alveoli were lined mostly by squamous type I pneumocytes and a few large cuboidal type II pneumocytes and inflated with thin inter-alveolar septa (Fig. 1A). Meanwhile, rats injected with DMBA showed undifferentiated tumor mass typically small cell carcinoma which is characterized by a high nuclear to cytoplasmic ratio, usually no nucleoli, and the cells have deeply basophilic molding nuclei and scanty cytoplasm along with characteristically grow bronchi with fine stroma, besides desquamation of bronchial epithelial, infiltration of malignant cells to submucosa and indentation of the cells due to apparent pressure from adjacent cells (Fig. 1B&C). Conversely, in treatment either with ROSA or cisplatin, the neoplastic masses were scattered along lung lobules without clear demarcation from surrounding tissues. Apoptosis of neoplastic cells was seen. Multifocal areas of emphysema with thickening of alveolar septa were seen. The tumor mass consisted of pleomorphic cells with deeply basophilic indented nuclei and scanty cytoplasm which separated by delicate fibrous stroma. Moreover, the responsibility of cancerous mass appeared as Grade Ib, morphologic evidence of therapy-induced changes with > 10% residual tumor (Fig. 1D&E). Additionally, the combination of ROSA & cisplatin exhibited shrinkage of tumor mass, regression of neoplastic cells infiltration of the bronchial wall, perivascular oedema and focal emphysematous areas compared with cisplatin or ROSA alone. Furthermore, they showed apoptosis and nuclear pyknosis of neoplastic cells with extensive response (Grade II a) with residual tumor *≤* 10% (Fig. 1F).

### Effect of ROSA and cisplatin alone or in synergy on poly (ADP-Ribose) Polymerase-1 (PARP-1)

**Fig. 2:**
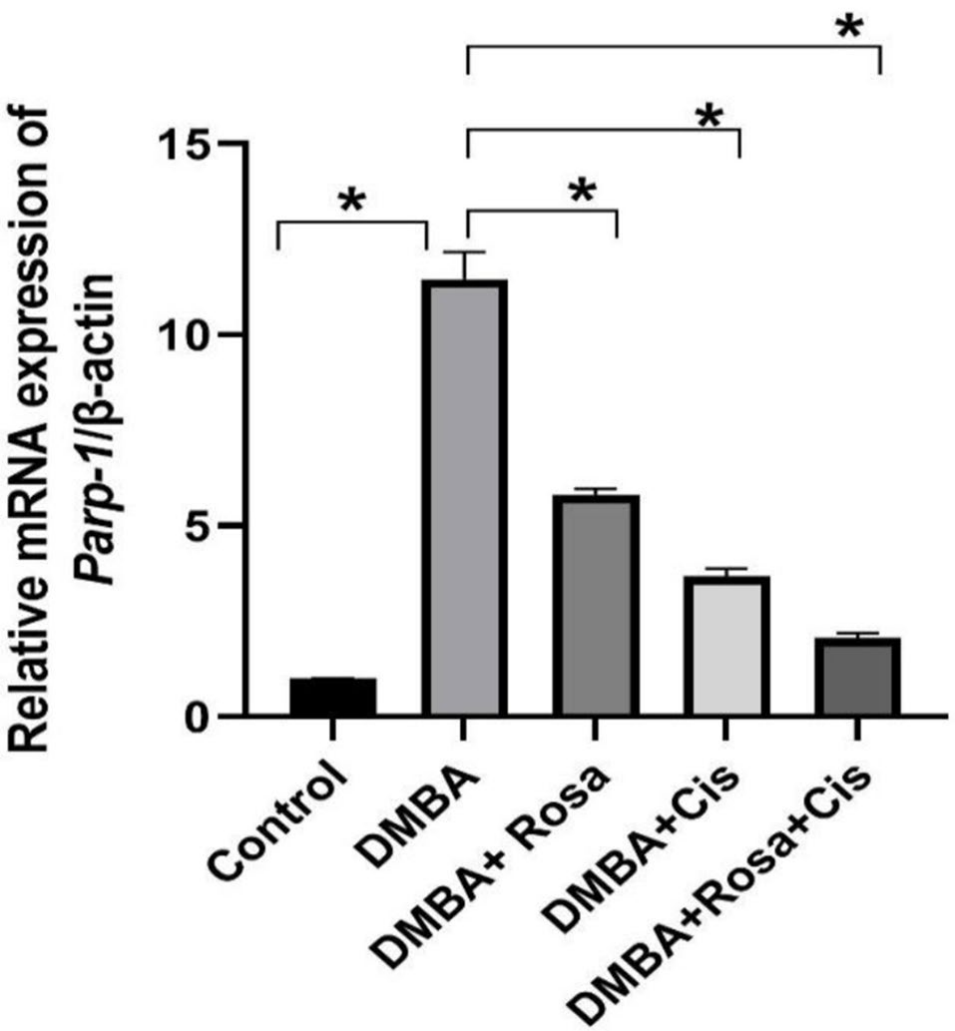
The effect of ROSA and cisplatin on the mRNA expression of *Parp-1*. Statistical data are expressed as mean ± SEM and analyzed by one-way ANOVA followed by Bonferroni test (* *P* < 0.01)

Regarding its mechanism of action, DMBA induces carcinogenesis through the excessive production of reactive oxygen species (ROS) and insertion of adenine and guanine nucleotides to DNA triggering DNA adducts and chromosomal aberrations promoting mutations and genomic instability which ultimately results in abnormal replication and carcinogenesis. Despite its role as a sensor and regulator molecule in DNA damage response (DDR) that mediates DNA repair, various studies reported its overexpression and upregulation in several diseases and cancer types. Herein, a notable overexpression of the *Parp-1* transcript in the lung tissues of rats injected with DMBA was observed compared to the normal rats. Thus, suggesting a defective and dysfunctional DNA repair system associated with carcinogenesis (Fig. 2). However, treatment with rosa and cisplatin either alone or in synergy downregulated the *Parp-1* transcript expression.

### Effect of ROSA and cisplatin alone or in synergy on TLR2/MyD88/ TRAF6/NF-κB signal pathway in lung

**Fig. 3:**
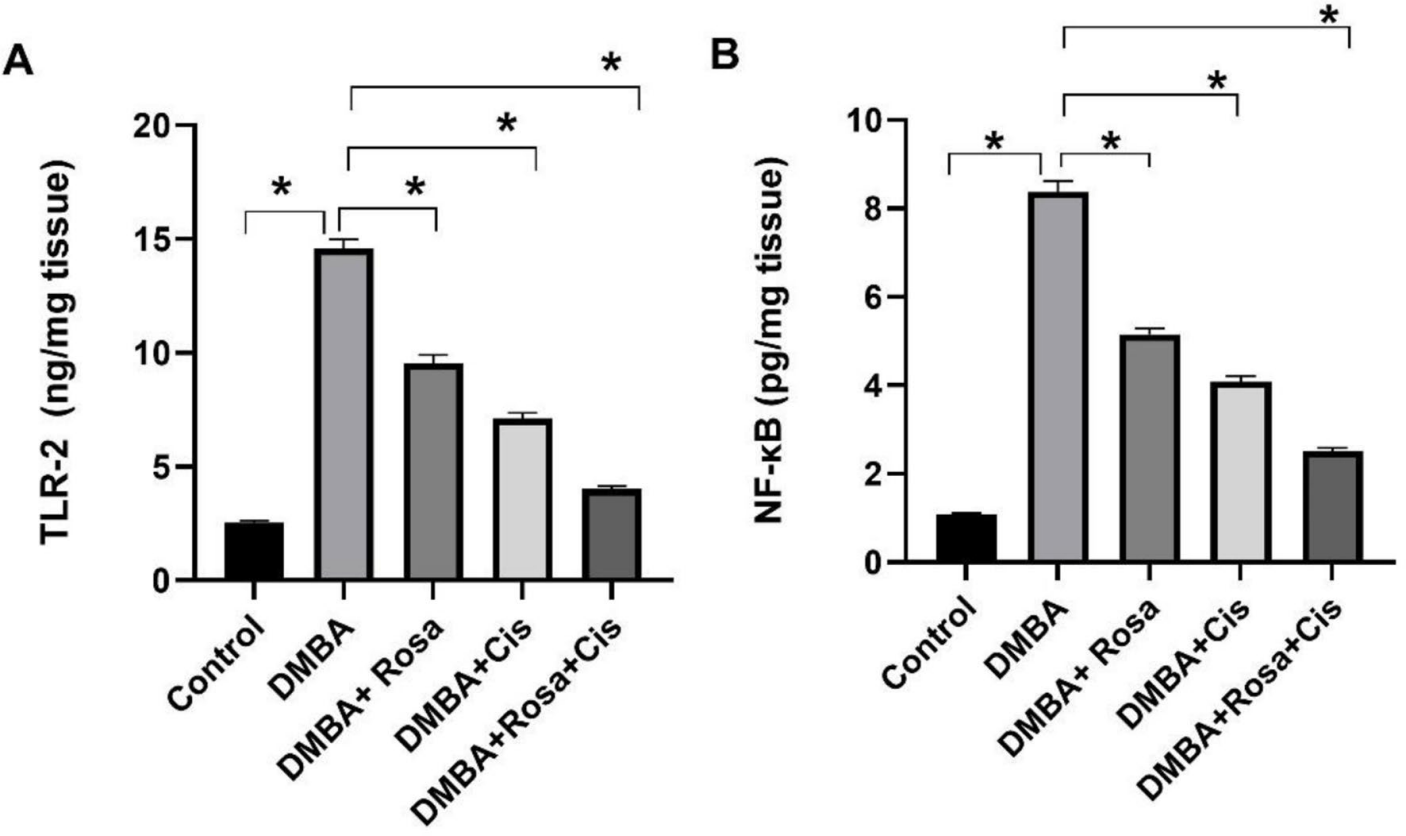
The effect of ROSA and cisplatin on the levels of TLR2 (**A**) and NF-κB (**B**). The statistical data are expressed as mean ± SEM analyzed by one-way ANOVA followed by Bonferroni test (* *P* < 0.01)

**Fig. 4:**
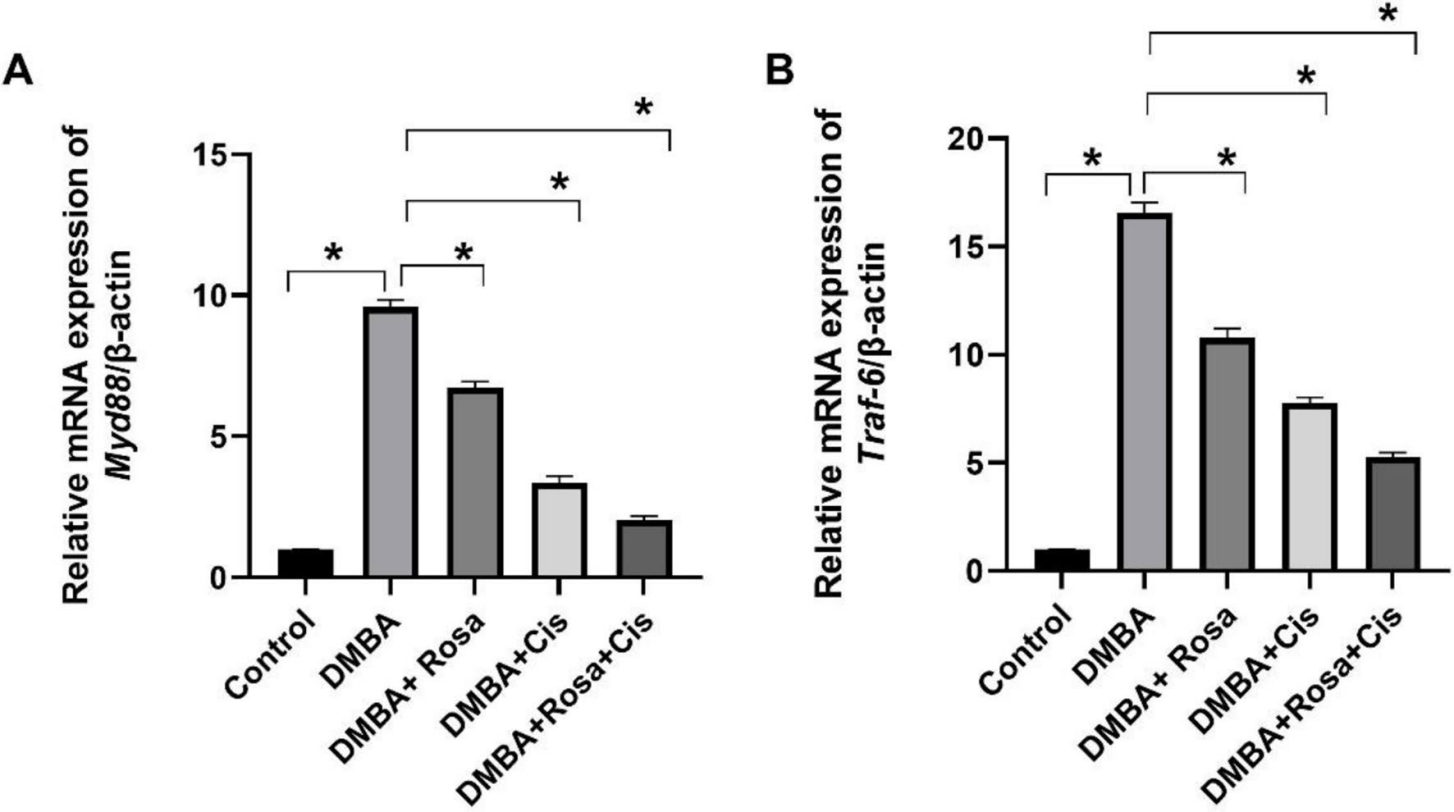
The effect of ROSA and cisplatin on the mRNA expression of *Myd88* (**A**) and *Traf6* (**B**). The statistical data are expressed as mean ± SEM analyzed by one-way ANOVA followed by Bonferroni test (* *P* < 0.01)

Owing to the potential role of TLR2 and its downstream signaling pathway (MyD88/ NF-κB besides TRAF6) in immunity, inflammation and carcinogenesis, their gene expression was explored. The results in Fig. 3 exhibited a marked increase in the levels of TLR2 and its downstream NF-κB in lung tissues of rats injected with DMBA, boosting lung cancer development. Consequently, the elevated levels of TLR2 were associated with upregulated expression of *Myd88 and Traf6* genes relative to the control group (Fig. 4). The binding of TLR2 to the adapter protein MyD88 recruits TRAF6 which consequently promotes the activation of the NF-κB Thus, the activation of this pathway may enhance carcinogenesis and development of lung cancer in addition to regulating cancer proliferation. Conversely, significant suppression of the TLR2/MyD88/ TRAF6/NF-κB signal pathway was noticed after treatment with rosa and cisplatin either alone or in combination.

### Effect of ROSA and cisplatin alone or in synergy on c-MET and PI3K/AKT/mTOR signal pathway in lung

**Fig. 5:**
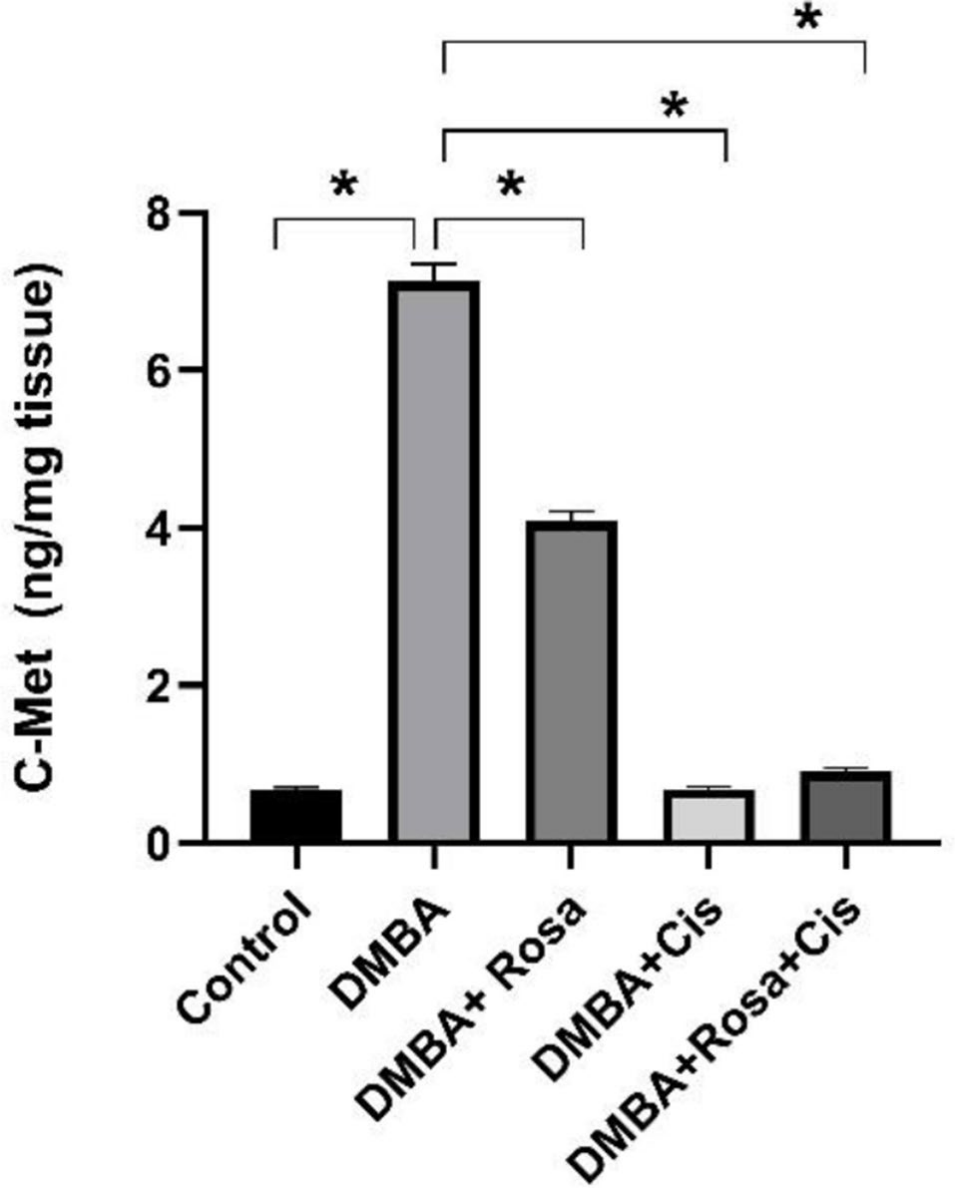
The effect of ROSA and cisplatin on the levels of c-MET. The statistical data are expressed as mean ± SEM analyzed by one-way ANOVA followed by Bonferroni test (* *P* < 0.01)

**Fig. 6:**
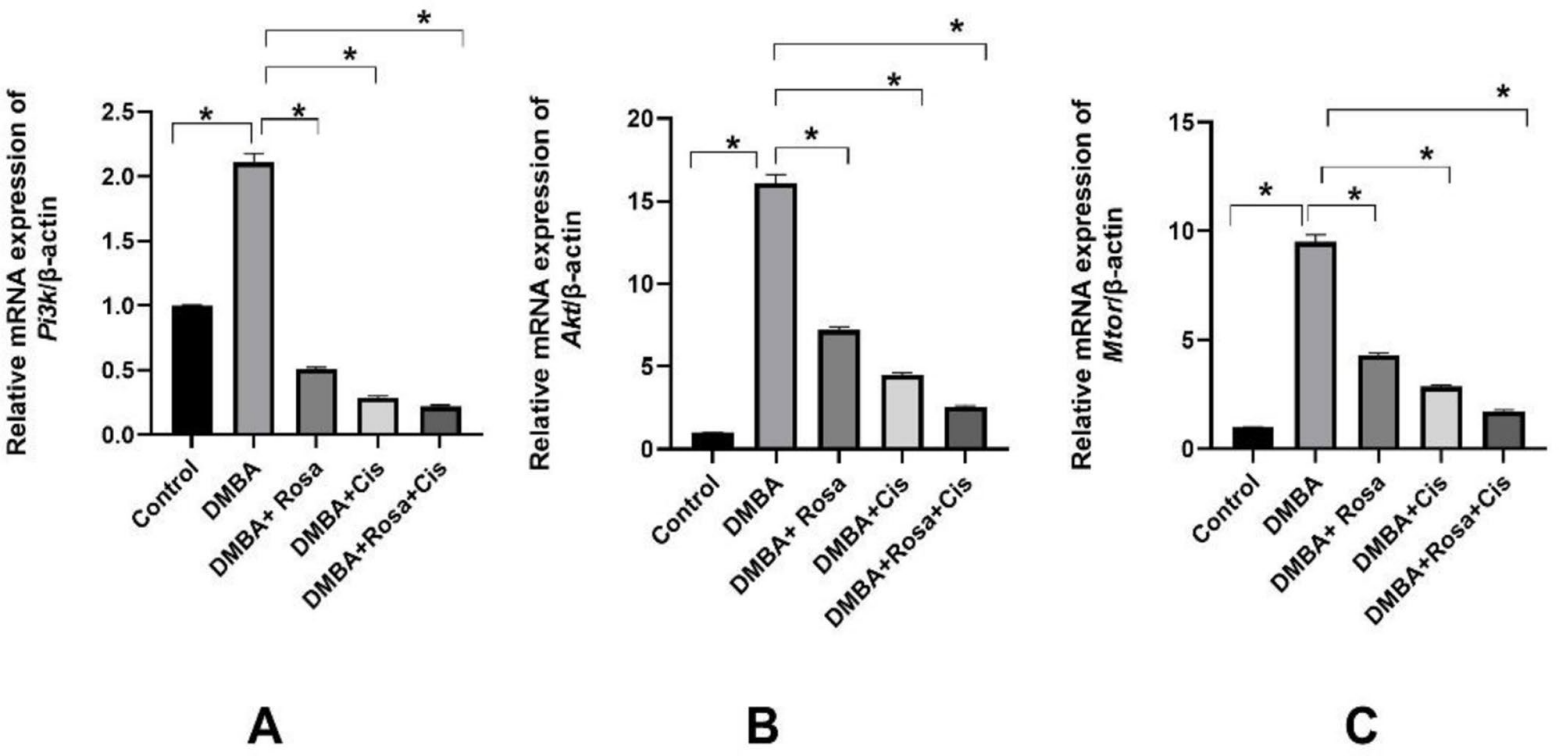
The effect of ROSA and cisplatin on the mRNA expression of *Pi3k* (**A**), *Akt* (**B**) *and Mtor* (**C**). The statistical data are expressed as mean ± SEM analyzed by one-way ANOVA followed by Bonferroni test (* *P* < 0.01)

Hepatocyte growth factor (HGF)/ cellular-mesenchymal-epithelial transition factor (c-MET) signaling is critical for the regulation of various normal physiological and biological processes such as embryonic development, wound healing and tissue regeneration. The constitutive and aberrant activation of c-MET mediated by NF-κB contribution provokes several downstream signaling pathways (MAPK, PI3K/AKT, STAT3) which subsequently promote cancer proliferation, survival, progression, angiogenesis, invasion and metastasis. Accordingly, the level of c-MET was detected by ELISA whereas the gene expressions of Pi3k, Akt, and Mtor were determined by RT-PCR in the lung tissues. As shown in Figs. (5&6), a dramatic elevation in the protein levels of c-MET coupled with an obvious upregulated mRNA expression of *Pi3k*, *Akt*, *and Mtor* in the cancerous lung tissues compared to the control indicates that the activation of the c-MET/ PI3K/AKT/mTOR exacerbating cancerous cell proliferation and progression. On the other hand, rosa and cisplatin inhibited lung cell proliferation by decreasing the levels of c-MET and downregulating the *Pi3k*, *Akt*, *and Mtor* transcript.

### Effect of ROSA and cisplatin alone or in synergy on Galectin-1 (Gal-1)

**Fig. 7:**
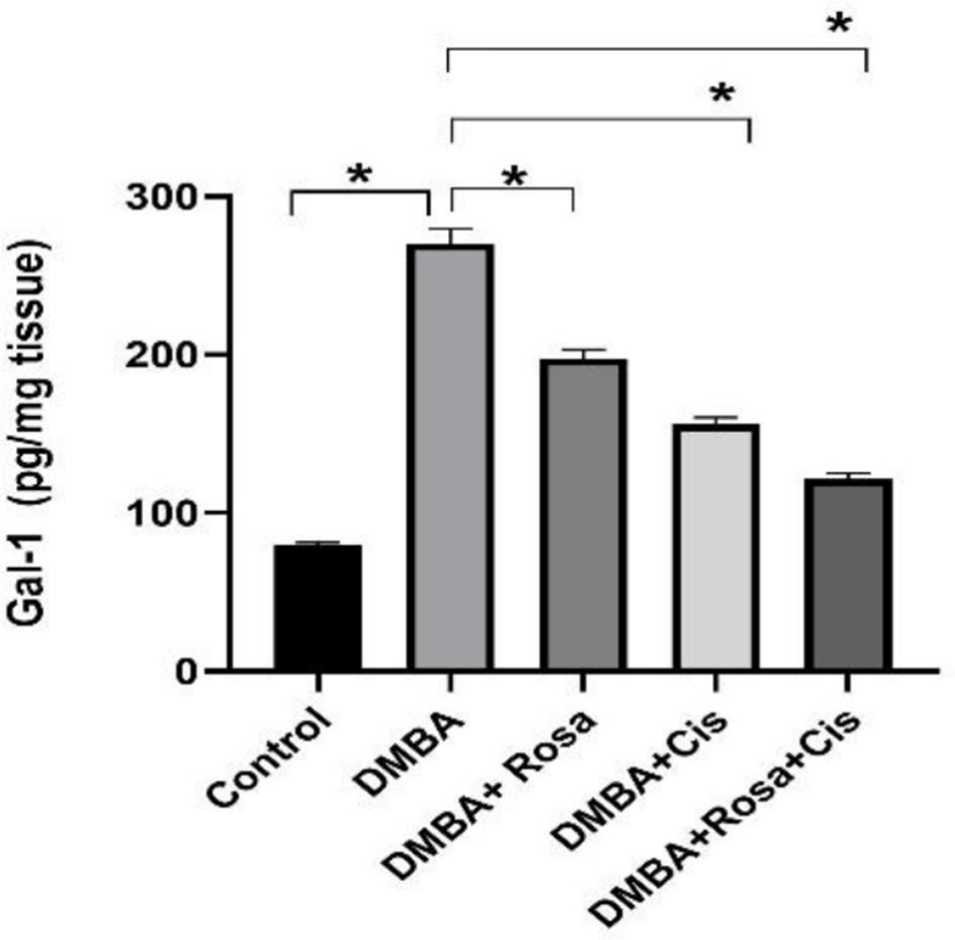
The effect of ROSA and cisplatin on Galectin-1 (Gal-1). The statistical data are expressed as mean ± SEM analyzed by one-way ANOVA followed by Bonferroni test (* *P* < 0.01)

Moreover, as shown in Fig. 7a considerable elevation in the levels of the Gal-1 protein was observed in the lung tissues of the DMBA group relative to that of the control. This confirms its tumorigenesis potential via promoting lung cell proliferation, progression, survival, and metastasis. In contrast, treatment with rosa or/ and cisplatin reduced the Gal-1 levels.

### Effect of ROSA and cisplatin alone or in synergy on autophagy

**Fig. 8:**
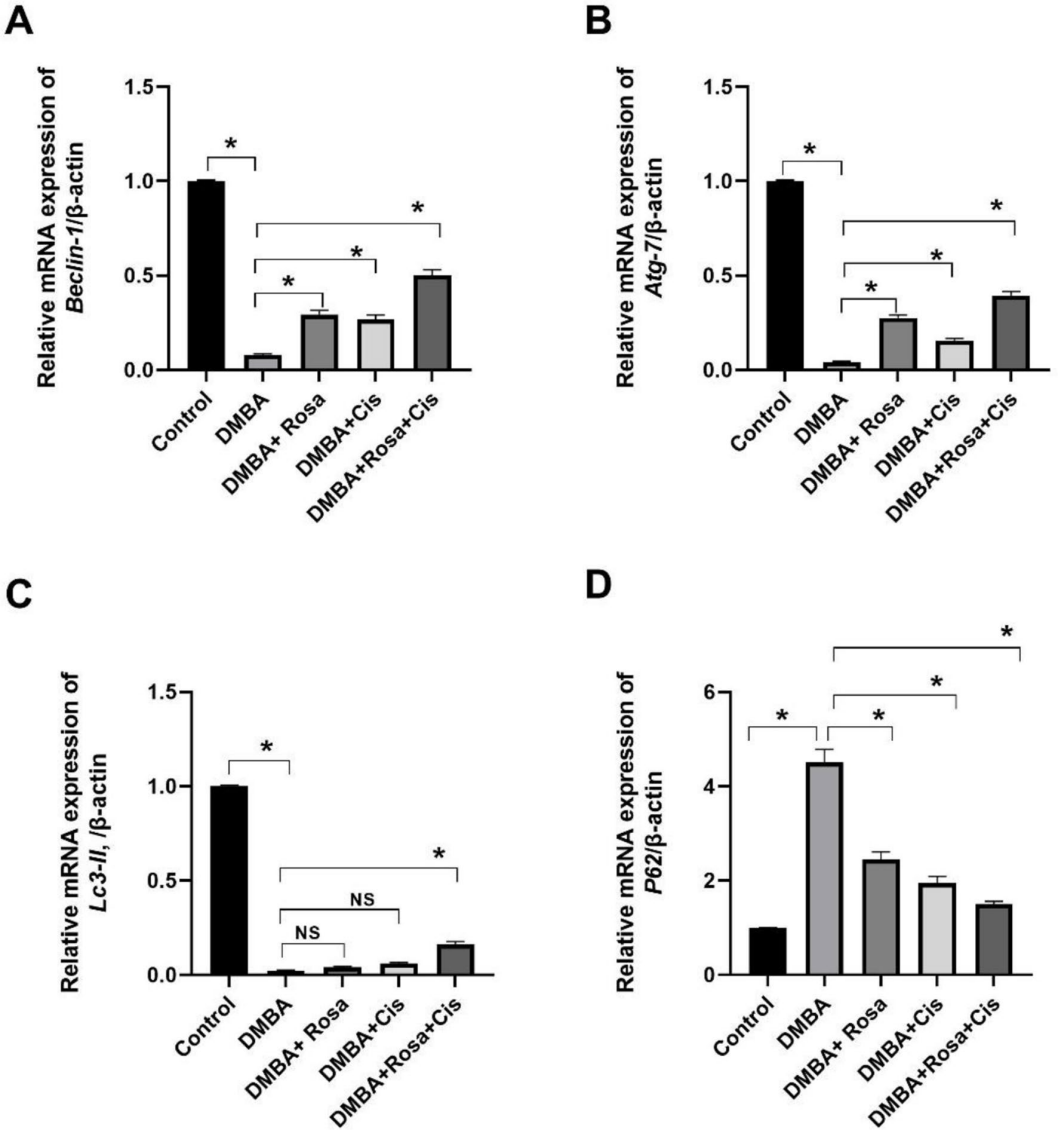
The effect of ROSA and cisplatin on autophagy. the mRNA expression of *Beclin-1* (**A**), *Atg-7* (**B**), *Lc3II* (**C**) and *P62* (**D**). The statistical data are expressed as mean ± SEM analyzed by one-way ANOVA followed by Bonferroni test (* *P* < 0.01). NS: non-significant

Interestingly, the obtained results showed that the promotion and progression of lung carcinogenesis besides the activation of the PI3K/AKT/mTOR pathway (a negative regulator of autophagy) were associated with the suppression of autophagy which has a regulatory effect on intracellular hemostasis, antitumor and apoptosis. Accordingly, as shown in Fig. 8, a remarkable decrease in the mRNA expression of Beclin-1, Atg-7 and Lc3II was accompanied with increased expression of the P62 transcript in the lung of rats injected with DMBA compared to control rats. On the contrary, Rosa and cisplatin treatment significantly elevated the mRNA expression of autophagic molecules (Beclin-1, Atg-7 and Lc3II) and inhibited that of the P62 gene, therefore inducing autophagy.

### Effect of ROSA and cisplatin alone or in synergy on apoptosis

**Fig. 9:**
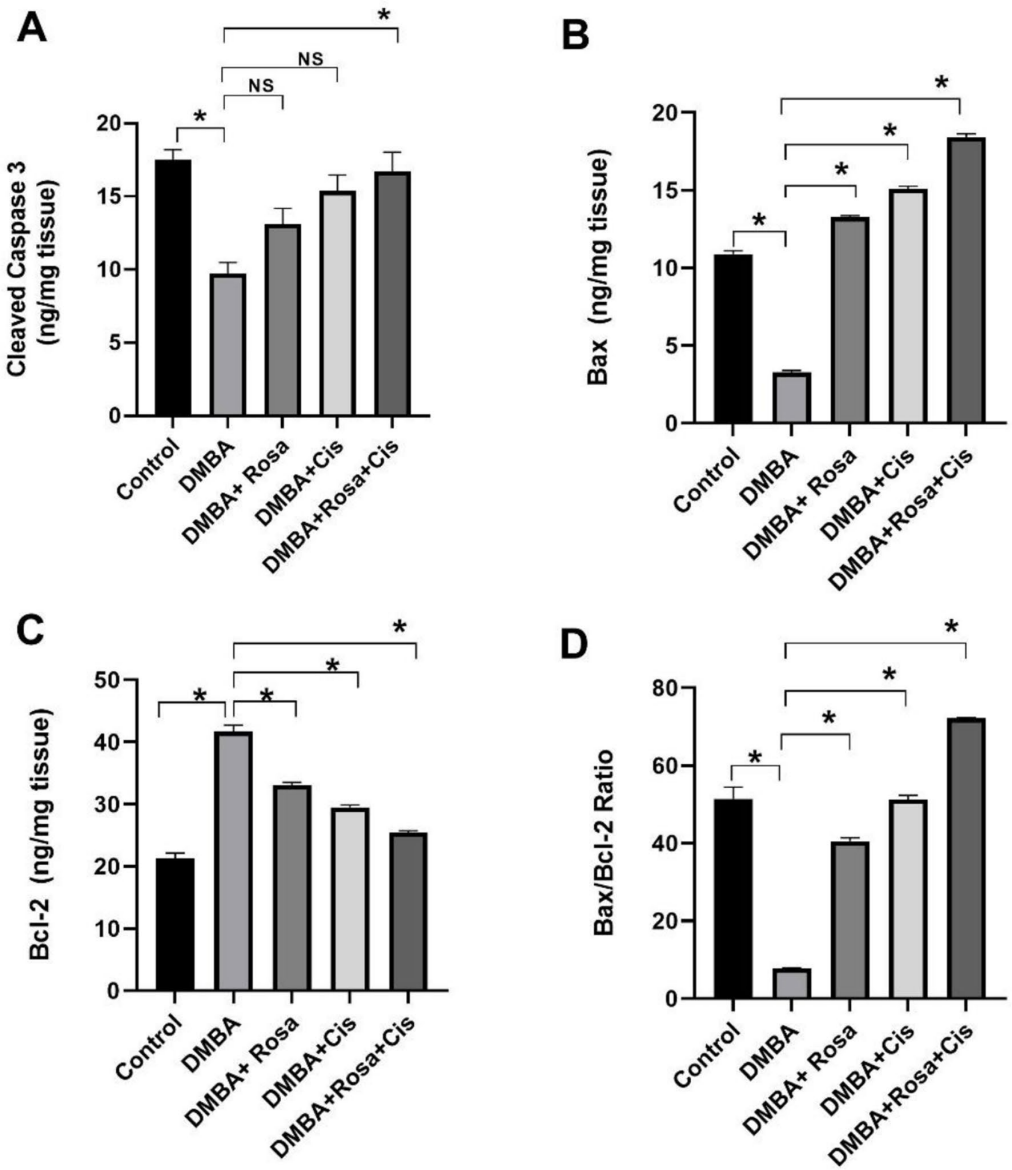
The effect of ROSA and cisplatin on apoptotic markers. Levels of caspase 3 (**A**), Bax (**B**), Bcl-2 (**C**) and Bax/Bcl-2 ratio (**D**). The statistical data are expressed as mean ± SEM analyzed by one-way ANOVA followed by Bonferroni test (* *P* < 0.01). NS: non-significant

Simultaneously, the improper activation of PI3K/AKT/mTOR, c-MET, Gal-l and NF-*κ* B implies higher proliferation activity of the cancer cells, dysregulated autophagy and hinders apoptosis. As shown in Fig. 9, it was found that DMBA injection produced a notable increase in the levels of the antiapoptotic protein Bcl-2 coupled with a drastic decline in the proapoptotic markers levels BAX and Caspase-3 as well as Bax/Bcl-2 ratio in the lung tissues relative to their control. As a result, carcinogenesis is facilitated while apoptosis is reduced. Meanwhile, treatment with rosa and cisplatin alone or in combination resulted in a significant decrease in Bcl-2 levels, as well as an increase in BAX, Caspase-3, and the Bax/Bcl-2 ratio.

## Discussion

The current study was designed to evaluate the effect of Rosa in synergy with cisplatin against lung cancer induced by DMBA. It has been previously found that DMBA produces ROS, forms DNA adducts triggers mutations and chromosomal aberrations and DNA damage leading to abnormal replication. It also activates many signaling pathways, such as PI3K/Akt, Wnt pathway, and NF-κB pathway which has a critical role in cell proliferation, survival, and invasion in various tissues through the covalent binding of its metabolites to DNA [34, 40]. The highly cytotoxic lesions and DNA breaks and damage induced by DMBA [41] disrupt the DNA repair system, initiate carcinogenesis [42] and activate PARP-1 [43, 44]. Parallel to the current results, previous studies reported a high expression of PARP1 in several malignancies including all subtypes of lung cancer especially squamous cell and lung adenocarcinomas enhancing lung cancer survival and metastasis [45, 46]. Meanwhile, treatment with rosa and cisplatin either alone or in synergy reduced the PARP-1 expression and it was found that its inhibition promotes the death of the cancer cells [42]. Previous studies indicated that hampering PARP1 activity reduces its DNA repair function, accumulating DNA single and double-strand breaks and increasing cell damage which eventually promotes cell death signaling pathways [47, 48]. PARP1 inhibition has a potential effect during cancer treatment via trapping the PARP1 molecule to DNA damaged site thus, more cytotoxicity [45].

Considerably, the current results showed a remarkable upregulated expression of the TLR2 and its downstream signal pathway MyD88, TRAF6 and NF-κB in lung tissues of the DMBA group relative to their control. It was found that TLR2/MyD88/NF-κB signal pathway activation via the TRAF6-TAK1 signaling axis is expressed in various malignancies including lung cancer [49]. Moreover, it exhibits a pro-tumor inflammatory potential [50], through the promotion of cancer proliferation, survival, progression and invasion [12, 51]. Feng et al. [52] demonstrated that the aberrant overexpression of the TRAF6 in lung cancer tissues triggered activation of AKT thus, boosting cancer proliferation and progression in non-Small Cell Lung Cancer (NSCLC). Furthermore, the overexpression of NF-κB in lung cancer potentiates its development and progression by promoting the expression of the anti-apoptotic Bcl-2 family and suppressing that of pro-apoptotic proteins, thus promoting proliferation and preventing apoptosis [53].

Conversely, treatment with rosa and cisplatin either alone or in combination significantly suppressed the TLR2/MyD88/ TRAF6/NF-κB signal pathway. In harmony with this, phytochemicals can enhance the susceptibility of cancer cells and animal tumor models to anticancer treatments by interfering with many processes, such as cell cycle arrest, DNA damage, angiogenesis, and variant signaling pathways, especially TLR/NF-κB/NLRP [18]. Moreover, Zhao et al. [12] indicated that inhibition of the TLR4/MyD88/NF-κB pathway has an anti-tumor effect and suppresses lung cancer growth. Additionally, Wang et al. [54] found that ginsenoside Rg3 amplified cisplatin efficacy in the lung cancer cell lines by inhibiting the NF-κB pathway.

Various studies indicated the cross-talk between c-MET and NF-κΒ whereas it was found that NF-κΒ activation contributed to the activation and induction of c-MET [55–57]. Our results are consistent with those of Miranda et al. [58] and Yu et al. [59] who confirmed the overexpression of the c-Met in lung cancer (adenocarcinoma and squamous cell carcinoma), and it was highly expressed in 61% of NSCLCs. The aberrant activation of c-MET following malignant transformation regulates many oncogenic processes like proliferation growth and progression, disrupting cell death, angiogenesis and metastasis [60]. Furthermore, it activates the PI3K/AKT/ mTOR signaling pathway and Bcl-2 signaling cascades that regulate cell survival [61, 62]. Additionally, it protects tumors and inhibits apoptosis [9]. Herein, a notable increase in the expression of the *Pi3k*, *Akt*, *and Mtor* transcript was found in the lung cancer tissues. This coincides with the finding of Sanaei et al. [63] who depicted that the dysregulated PI3K/Akt/mTOR pathway contributes to lung cancer promotion and progression. In contrast, rosa and cisplatin hindered lung cell proliferation by decreasing the levels of c-MET, and downregulating the expression of PI3K, AKT, and mTOR. Yu et al. [59] revealed that inhibition of the c-MET signaling pathway diminished the proliferation and metastasis and mediated apoptosis in lung cancer cells via suppressing the phosphorylation of its downstream PI3K/AKT/mTOR pathways. Moreover, using inhibitors for both c-Met and PARP synergistically have anti-tumor, and antiproliferative potential via disrupting tumor growth, suppressing invasion and promoting DNA damage which in turn triggers apoptotic cell death in lung cancer [64].

Owing to its pro-neoplastic role Gal-1 promotes tumor growth, development, progression and metastasis [65] as well as immune escape by tumors [66]. In line with our results, a notable overexpression of galectin-1 levels was observed in lung cancer tissues especially adenocarcinoma which promotes lung cancer progression, invasion migration and metastasis [67, 68]. Zhou et al. [69] revealed that Gal-1 promoted lung tumorigenesis and invasiveness by AKT activation. However, the knockdown and decreasing the levels of Gal-1 considerably delayed lung cancer growth and diminished cancer migration, invasion and metastasis [70, 71]. Furthermore, Su et al. [72] indicated that Gal-1 inhibition enhanced the sensitivity of the hepatocellular carcinoma cells toward cisplatin and augmented its anti-tumor activity.

The interplay between autophagy and apoptosis maintains cellular homeostasis while any perturbation in this dynamicity endorses uncontrolled cell growth and carcinogenesis [73] and contributes to lung cancer progression and pathogenesis [74]. Both are closely interconnected and regulated by different signaling pathways [75]. Autophagy is usually regulated by PI3K /AKT and its downstream mTOR which negatively inhibits autophagy [76]. It was exhibited that suppression of autophagy was associated with overexpression expression and abnormal increase of p62 in cancerous tissues [77] which is inversely correlated with Beclin 1 expression in NSCLC tissues [78]. Moreover, the interaction of Bcl-2 with Beclin-1 or Bax suppressed both autophagy and apoptosis and potentiated the anti-apoptotic role [79, 80].

The current results showed that rosa and cisplatin promoted autophagy and apoptosis by elevating levels of the autophagy markers (ATG-7, Beclin-1, and LC3-II) and the apoptotic markers (caspase-3 and Bax) with the inhibition of the P62 and Bcl-2 in addition to suppressing the expression levels of PI3K, Akt and mTOR thus inhibiting the PI3K/Akt/mTOR pathway. This was in line with the results of Wang et al. [81] who reported that inhibition of the PI3K/AKT/mTOR signaling pathway induced autophagy in tumor cells which was associated with cytoprotective apoptosis [82]. Additionally, Wu et al. [83] found that activated caspases amplified the apoptotic cell death by changing the autophagic protein fragments into pro-apoptotic fragments, in which caspase-3 cleaves Beclin-1 leading to its translocation to mitochondria and the release of cytochrome C triggering apoptosis [84].

Various previous studies exhibited the anti-cancer effects of *Rosa canina* extract due to its anti-oxidant and anti-proliferative which was attributed to the presence of polyphenols. Tumbas et al. [85] reported that quercetin, ellagic acid and vitamin C are the most abundant antioxidant compounds in *R. canina* responsible for its antioxidant activity, while only polyphenols contribute to its cytotoxic activity. Cagle et al. [86] indicated that *R. canina* extract inhibited the proliferation of human glioblastoma cells by increasing cell cycle arrest at the G_2_/M phase and blocking both the MAPK and AKT signaling mechanisms. Moreover, Naseri et al. [21] showed that the antiproliferative effects of *R. canina* extract against thyroid cancer cells (B-CPAP AND THR.C1-PI 33) was attributed to induction of the apoptotic cell death pathway through increased Bax /Bcl2 proportion together with up-regulating p53 and Caspase 3 expression.

One of the limitations of this study is using only ELISA technique for protein detection. Therefore, further studies are required as an extensive in-depth analysis regarding the active forms of the studied proteins to obtain a convincing conclusion and emphasize the exact mechanism of the anticancer mechanism of Rosa in the future along with this study.

## Conclusion

In conclusion, the current results showed that Rosa synergistically improves the chemotherapeutic potential of cisplatin in lung cancer cells by suppressing the TLR2/MyD88/ TRAF6/NF-κB signal pathway together with the PI3K/AKT/mTOR and Gal-1 which consequently, inhibits proliferation. Moreover, these treatments not only inhibited the expression of both c-MET and PARP-1 but also remarkably elevated the levels of autophagy markers (ATG-7, Beclin-1, and LC3-II) and the apoptotic markers (caspase-3 and Bax) with the inhibition of the P62 and Bcl-2 which collectively, boosts autophagy and apoptotic cell death of lung cancer cells. Overall, the combined treatment with Rosa and cisplatin is more effective than each alone. Thus, Rosa may act as chemosensitizer and potentiate cisplatin effectiveness and mitigate its adverse effects. However, further studies are still needed to investigate the exact anticancer mechanism of Rosa and whether it can be used as a novel therapeutic agent for cancer treatment.

## Data Availability

All data obtained from this study are included in the current manuscript.
